# Differential Effects of Collagen Hydrolysate Digesta on Osteoclast and Osteoblast Responses In Vitro

**DOI:** 10.3390/nu18162688

**Published:** 2026-08-18

**Authors:** Sirui Shan, Christina E. Larder, Josephine T. Tauer, Michèle M. Iskandar, Svetlana V. Komarova, Stan Kubow

**Affiliations:** 1 School of Human Nutrition, McGill University, Ste-Anne-de-Bellevue, QC H9X 3V9, Canada; sirui.shan@mail.mcgill.ca (S.S.); christina.larder@genacol.ca (C.E.L.); michele.iskandar@mcgill.ca (M.M.I.); 2 Corporation Genacol Canada Inc., Blainville, QC J7C 6B4, Canada; 3 Faculty of Dental Medicine and Oral Health Sciences, McGill University, Montréal, QC H3A 1G1, Canada; josephine.tauer@jku.at (J.T.T.); komarova@ualberta.ca (S.V.K.); 4 Shriners Hospitals for Children-Canada, Montréal, QC H4A 0A9, Canada; 5 Department of Pediatrics and Adolescent Medicine, Johannes Kepler University, 4020 Linz, Austria; 6 Department of Biomedical Engineering, Faculty of Engineering, University of Alberta, Edmonton, AB T6G 1H9, Canada

**Keywords:** osteoarthritis, collagen hydrolysate, bioactive peptides, osteoclast, osteoblast, bone remodeling, nutraceutical

## Abstract

**Background/Objectives**: Osteoarthritis (OA) is partly driven by an imbalance between bone-resorbing osteoclasts (OCs) and bone-forming osteoblasts (OBs). Collagen hydrolysates (CHs) are widely used for OA symptom management, with potential benefits attributed to bioactive peptides (BAPs) released during digestion. However, their direct effects on bone cells remain unclear. **Methods**: Two bovine-sourced CHs, CH-GL and CH-OPT, were digested in vitro and tested on primary murine OC and OB cultures. Osteoclastogenesis was evaluated mainly under standard RANKL conditions (50 ng/mL), with exploratory analysis under high RANKL conditions (100 ng/mL). OC cultures were exposed to CH digesta at 0.01, 0.05, 0.1, or 0.5 mg/mL, while OB cultures were exposed to 0.01 or 0.1 mg/mL. **Results**: Under standard RANKL conditions, CH-GL significantly reduced OC area by over 50% across all tested concentrations, while OC number was not significantly altered. This coincided with decreased expression of selected osteoclastogenic markers, including *Nfatc1*, *Ctsk*, *Dc-stamp*, and *Oscar*. In OB cultures, CH-GL increased *Runx2*, *Osterix*, and *Alp* expression at 0.1 mg/mL and reduced *Mmp9* expression at both tested doses. It also modestly increased mineralization-related signal intensity and collagen-associated matrix area. For CH-OPT, selected reductions in *Dc-stamp* and *Oscar* expression were observed, while OC number, OC area, and most OB-related outcomes were not significantly altered. **Conclusions**: CH digesta modulated selected OC- and OB-related outcomes in primary murine cells. These preliminary in vitro findings support further investigation of CH-derived bioactives in models that more closely reproduce OA-associated joint pathology.

## 1. Introduction

Osteoarthritis (OA) is the most common form of arthritis, and with a global aging population, the social and financial burden of OA is expected to rise significantly [1]. Patients with OA often experience joint deformity, swelling, pain, and reduced mobility [1,2]. Although OA was traditionally considered a “wear and tear” disease associated with cartilage degeneration, it is now recognized as a condition affecting the entire joint [3]. Notably, the subchondral and underlying trabecular bone play a critical role in the onset, progression, and severity of OA [3]. Although the exact crosstalk mechanisms remain incompletely understood, disruptions in bone remodeling are commonly associated with cartilage defects [3].

Despite progressive deterioration of cartilage and bone structure in OA, no approved disease-modifying drugs currently halt or slow the progression of the disease [1]. Consequently, OA patients often turn to nutritional supplements and nutraceuticals for symptom management [2]. A commonly used supplement is collagen hydrolysate (CH), which has demonstrated positive clinical outcomes, including reduced joint pain and improved joint comfort and mobility [2]. These benefits are thought to be partly related to bioactive peptides (BAPs) released from CHs during gastrointestinal (GI) digestion [4]. Consistent with this, collagen-derived peptides have been detected in blood and plasma following oral CH ingestion [4]. Bioavailable BAPs, such as prolyl-hydroxyproline (Pro-Hyp), can be absorbed and have also been detected in cartilage and bone cells [4,5].

Given the integral role of subchondral bone in OA pathogenesis, it is crucial that collagen-derived products not only provide symptomatic relief but also exert beneficial effects on bone remodeling. Indeed, previous preclinical studies and human clinical trials have demonstrated beneficial effects of collagen products on bone health. Specifically, in vitro and in vivo preclinical studies indicate that collagen supplementation can reduce bone loss, enhance bone strength, and improve overall bone biomechanical properties [6,7,8,9,10,11]. Corroborating these findings, human clinical trials and recent meta-analyses consistently report that collagen supplementation effectively increases bone mineral density and improves bone metabolism markers [4,12,13].

Bone remodeling is a dynamic process tightly regulated by bone-resorbing osteoclasts (OCs) and bone-forming osteoblasts (OBs) [14,15]. Osteoclastogenesis, the differentiation of hematopoietic stem cells into mature OCs, is primarily regulated by the receptor activator of nuclear factor kappa-B (RANK)/RANK ligand (RANKL) pathway [16]. RANKL, secreted by OBs, binds to the RANK receptor on OC precursors, promoting their differentiation and fusion into mature multinucleated OCs [16].

To date, studies assessing the effects of CHs on bone remodeling have more extensively examined OB-related outcomes than OC-related responses [17], although the mechanisms of action of CHs remain unclear. Previous work using collagen-derived peptides in MC3T3-E1 pre-osteoblast cells showed increased expression of OB differentiation-related genes, such as runt-related transcription factor 2 (*Runx2*) [18]. Similarly, treatment of MC3T3-E1 cells with the collagen-derived dipeptide Pro-Hyp increased alkaline phosphatase (ALP) activity and upregulated the expression of *Runx2*, Sp7 transcription factor (*Osterix*), and *Col1a1*, supporting a role for collagen-derived peptides in OB differentiation [19]. Increased OB mineralization and collagen synthesis have also been reported following CH or collagen peptide treatment [4,20,21]. In contrast, data on CH effects in OCs remain limited, although some existing studies suggest that collagen treatments may influence OC differentiation, morphology, or function [6,7,9,15].

However, most previous in vitro studies applied intact CHs or selected collagen peptides directly to bone cell cultures [18,19,20,21], without accounting for GI digestion and hepatic first-pass metabolism. These processes generate specific bioavailable BAPs to which bone cells may be exposed in vivo [4].

Previously, we characterized the key peptide content of two commercially available bovine-sourced CHs, Genacol Original Formula^®^ (CH-GL) and Selection (CH-OPT), following simulated in vitro GI digestion [22] and hepatic first-pass metabolism [23]. Building on this previous work, the present study aimed to characterize the effects of CH-GL and CH-OPT digesta on OC- and OB-related outcomes in primary murine cells in vitro. OC responses were evaluated mainly under standard RANKL conditions (50 ng/mL), with exploratory analysis under high RANKL conditions (100 ng/mL) to assess responses under enhanced osteoclastogenic stimulation [3,14,16]. OC outcomes included OC number, OC area, and osteoclastogenic gene expression, while OB outcomes included osteogenic gene expression, matrix-associated staining, and mineralization-related staining.

## 2. Materials and Methods

**Figure 1:**
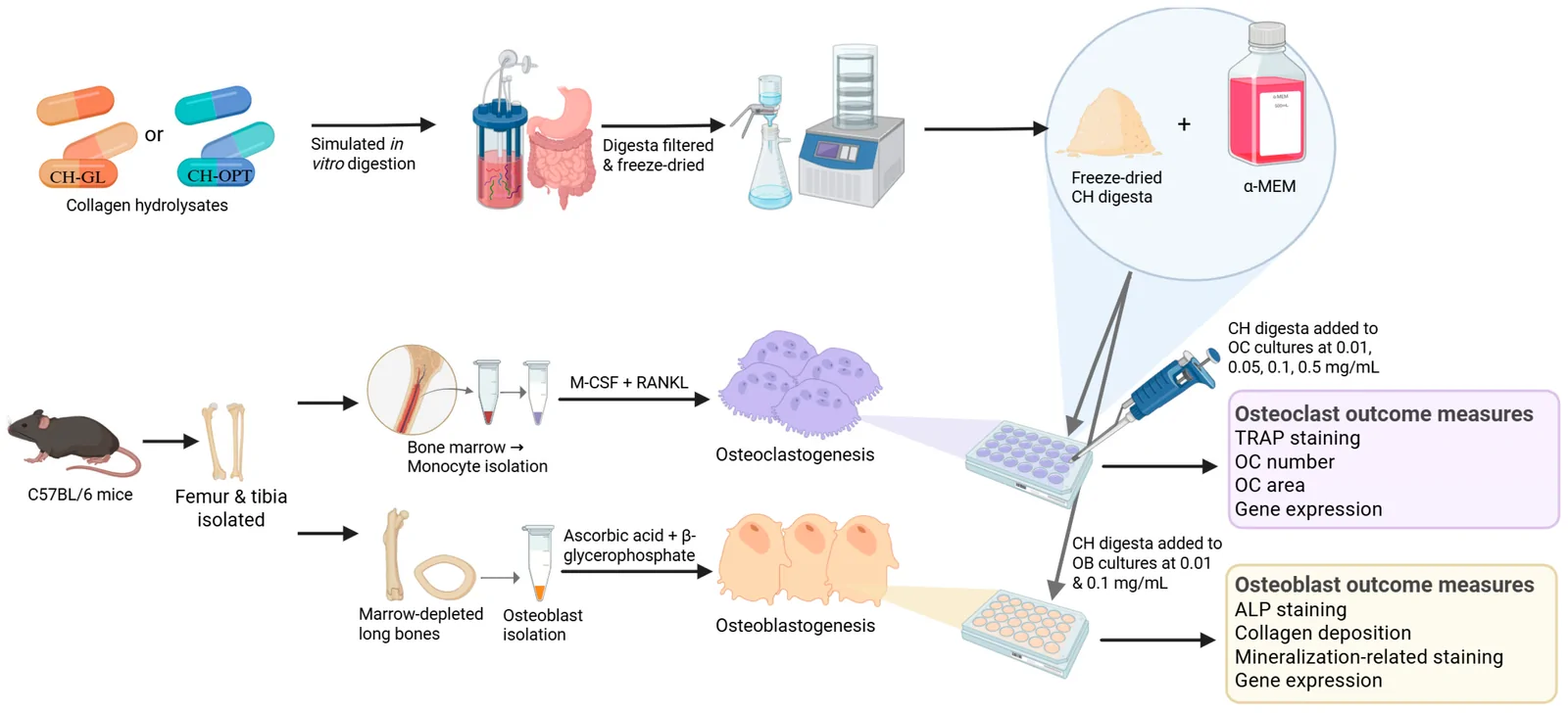
**Overview of the experimental design.** CH-GL and CH-OPT underwent simulated in vitro gastrointestinal digestion, 10 kDa filtration, and freeze-drying. The resulting CH digesta were reconstituted in α-MEM and applied to primary murine osteoclast (OC) and osteoblast (OB) cultures. Femora and tibiae from C57BL/6 mice were used to isolate bone marrow-derived monocyte/OC precursors and primary OBs from marrow-depleted long bones. Osteoclastogenesis was induced using M-CSF and RANKL, whereas OB cultures were maintained in osteogenic medium containing ascorbic acid and β-glycerophosphate. CH digesta were evaluated at 0.01, 0.05, 0.1, and 0.5 mg/mL in OC cultures and at 0.01 and 0.1 mg/mL in OB cultures. OC outcomes included TRAP staining, OC number, OC area, and gene-expression analysis. OB outcomes included ALP staining, collagen deposition, mineralization-related staining, and gene-expression analysis. Created in BioRender. Larder, C. (2026) https://BioRender.com/lipjq35.

The overall experimental design and workflow are summarized in Figure 1.

### 2.1. Collagen Hydrolysate Digestion and Treatment Concentration Selection

**Figure 2:**
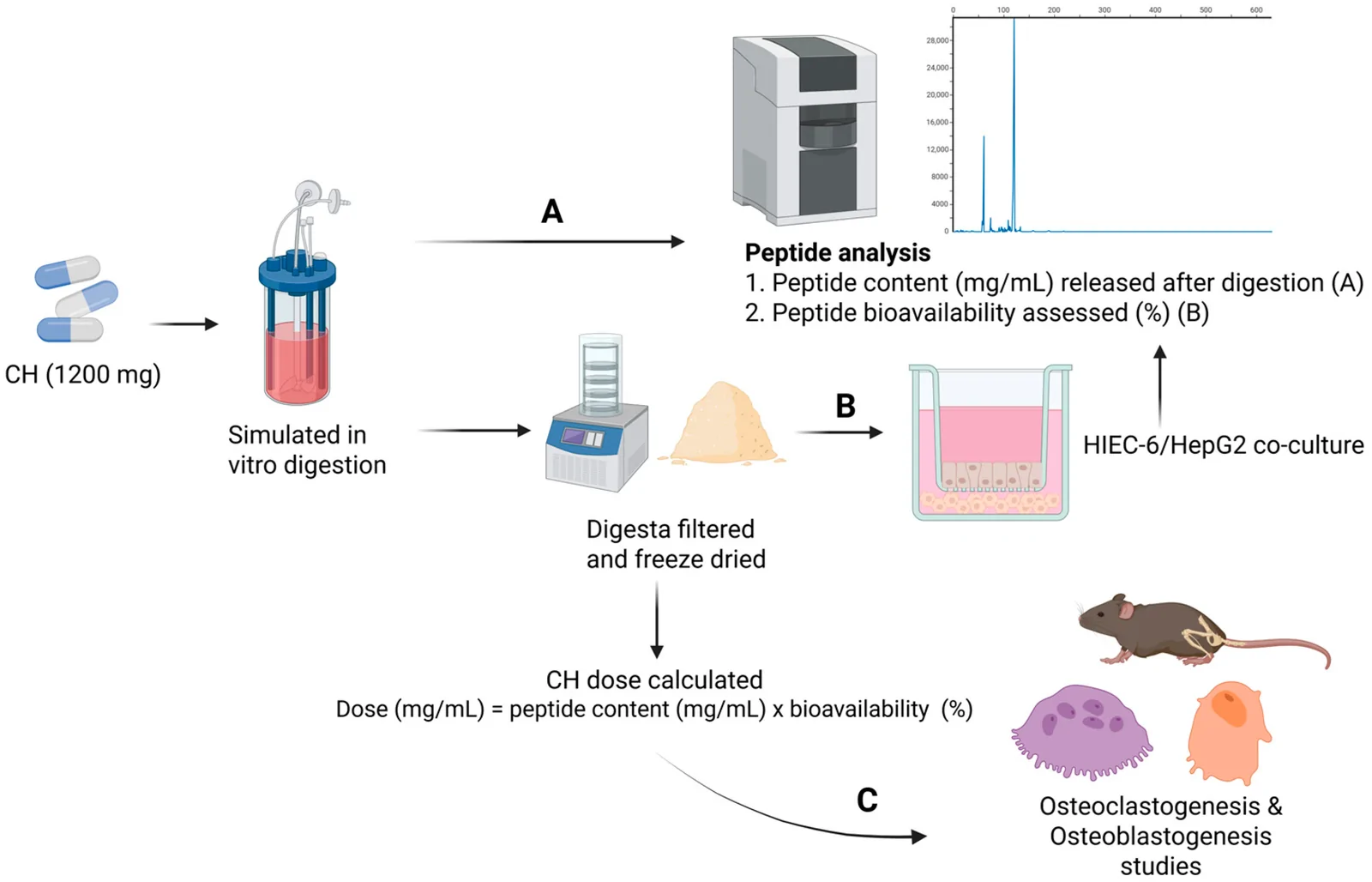
**Rationale for selection of CH digesta treatment concentrations.** (**A**) CHs underwent in vitro digestion followed by peptide content analysis [22]. (**B**) Filtered and freeze-dried CH digesta were applied to HIEC-6/HepG2 co-cultures to assess peptide bioavailability (%) following first-pass metabolism [23]. (**C**) Peptide content after digestion and bioavailability data (%) were used to determine doses of freeze-dried CH digesta for cell culture studies. Created in BioRender. Larder, C. (2026) https://BioRender.com/bihelgc.

In this study, the bovine-sourced CH products Genacol Original Formula^®^ (Genacol, Blainville, QC, Canada) (CH-GL) and Selection (Uniprix, Saint-Léonard, QC, Canada) (CH-OPT) were used. The specific bovine tissue source and detailed primary processing information were not available to the authors. Both CHs first underwent in vitro digestion [22] (Figure 2A; see Section 2.1.1 for details) followed by first-pass metabolism using a previously established HIEC-6/HepG2 co-culture model (Figure 2B) [23]. Peptide bioavailability (%) was then determined as described previously [23] (see Section 2.1.2 for details). For the present bone-cell experiments, however, the cells were treated with the <10 kDa filtered, freeze-dried digesta collected after in vitro digestion and before exposure to the HIEC-6/HepG2 co-culture. Bioavailability data from the HIEC-6/HepG2 model were used only to inform the selection of the experimental CH digesta concentrations applied to the OC and OB cultures (Figure 2C).

#### 2.1.1. In Vitro Digestion of Collagen Hydrolysate

Both CH products underwent simulated gastric and small intestinal digestion as described previously [23]. In brief, 1200 mg of each CH was digested with pepsin (4% *w*/*w*; #P7125, Sigma-Aldrich, St. Louis, MO, USA) for 30 min at pH 2.0, followed by pancreatin (4% *w*/*w*; #P7545, Sigma-Aldrich, St. Louis, MO, USA) for 1 h and 30 min at pH 7.5. Enzyme activity was stopped by the addition of NaOH until the pH reached 10, and the resulting digesta were filtered using an Amicon stirred ultrafiltration reactor (MilliporeSigma, Burlington, MA, USA) equipped with a membrane filter (molecular weight cut-off of 10 kDa) at 4 °C under a nitrogen gas pressure of 40 psi. This ultrafiltration step was used to collect the low-molecular-weight fraction and is expected to reduce the presence of intact digestive enzymes. Filtrates were freeze-dried at −50 to −60 °C and 0.85 mbar (0.64 mm Hg) using a Gamma 1–16 LSC freeze dryer (Martin Christ Gefriertrocknungsanlagen GmbH, Osterode am Harz, Germany) and stored at −80 °C until further use.

#### 2.1.2. CH Treatment Concentration Selection

Pro-Hyp was selected as a reference peptide because it is a well-characterized collagen-derived dipeptide, and because quantitative data on its post-digestion content and estimated bioavailability were available from our previous studies [22,23]. As no significant differences in Pro-Hyp content or bioavailability were observed between CH-OPT and CH-GL [22,23], data obtained for CH-GL were used as a conservative lower-end reference for selecting the treatment concentration range for both CH digesta.

In our prior work, the in vitro digestion of 1200 mg CH-GL, corresponding to the manufacturer-recommended daily human dose [24,25,26], yielded approximately 42 μg/mL Pro-Hyp [22]. Subsequent in vitro first-pass metabolism resulted in an estimated Pro-Hyp bioavailability of approximately 26% [23]. The estimated bioavailable Pro-Hyp concentration was calculated as follows:

Estimated bioavailable Pro-Hyp concentration = 42 μg/mL × 0.26 = 10.92 μg/mL(1)

This value was used only as a numerical reference for selecting 0.01 mg/mL as the lowest nominal CH digesta concentration. It was not a direct compositional conversion and does not imply that 0.01 mg/mL total digesta contained 0.01 mg/mL Pro-Hyp.

Because the estimate was based solely on Pro-Hyp, it did not account for other digestion-derived peptides, free amino acids, or other low-molecular-weight components present in the total digesta.

To provide a broader experimental concentration range while taking into account interindividual variability in absorption and metabolism [27], three additional doses (0.05, 0.1, and 0.5 mg/mL) were included. The selected range also encompassed concentrations used in previous mechanistic in vitro studies [7,15,20], while the broader oral-dose ranges evaluated in clinical studies [13,28,29] provided contextual support only and were not used to calculate the cellular treatment concentrations.

The freeze-dried CH digesta powders were weighed, dissolved in α-MEM with L-glutamine (#12000-022, Gibco, Thermo Fisher Scientific, Grand Island, NY, USA)) without additional supplements, filter-sterilized using a 0.22 µm filter, and diluted to final concentrations of 0.01, 0.05, 0.1, and 0.5 mg digesta/mL of culture medium before application to the cells. Accordingly, the applied concentrations represent nominal total-digesta dry-mass concentrations; the actual Pro-Hyp and total peptide concentrations in the specific treatment preparations were not measured.

### 2.2. Animals

All experimental procedures were approved by the Animal Care Committee of McGill University (protocol code SHRN7127, Approval Date: 1 July 2019), conducted in accordance with the ARRIVE guidelines, and complied with the ethical standards of the Canadian Council on Animal Care. A total of twelve 11-week-old C57BL/6 mice (9 males and 3 females) were used. OC precursors were obtained from eight male mice, whereas OBs were isolated from all twelve mice, as detailed in Section 2.3 and Section 2.5. The study was not designed to assess sex-specific effects, and the sex distribution was therefore unbalanced. The number and sex distribution reflected tissue availability and the successful isolation of sufficient viable primary cells. Cells derived from each animal were processed separately, with each animal constituting an independent biological replicate. Animals and their derived samples were tracked using coded identifiers for sample management. The C57BL/6 mouse colony was maintained at the Animal Care Facility of the Shriners Hospitals for Children-Canada. Mice were housed in groups of 3–5 sex- and genotype-matched littermates per cage under a 12 h light/dark cycle (6:00 AM–6:00 PM) at 21 °C, with *ad libitum* access to standard rodent chow (Rodent Chow #5075, Charles River Laboratories, Saint-Constant, QC, Canada) and water. Anesthesia was induced and maintained using inhaled isoflurane. Animals were euthanized by intracardiac puncture followed by cervical dislocation. Long bones were carefully dissected and cleared of surrounding soft tissue prior to further OC and OB isolation.

### 2.3. Osteoclast Isolation and In Vitro Osteoclastogenesis

OC precursors were obtained from the long bones of eight 11-week-old male mice, as described previously [30]. In brief, femora and tibiae were isolated and cleaned of soft tissue. Bone marrow was collected via centrifugation for monocyte/OC precursor isolation, and the remaining marrow-depleted long bones were used for OB isolation. Red blood cells (RBCs) were removed from the collected bone marrow using RBC lysing buffer (#R7757, Sigma-Aldrich, St. Louis, MO, USA), following the manufacturer’s protocol. Following RBC lysis, the reaction was stopped, and cells were washed with complete culture medium (see Supplementary Table S1). The resulting cell pellet was resuspended in complete culture medium supplemented with macrophage colony-stimulating factor (M-CSF, 50 ng/mL) and incubated overnight in a T-75 flask (Falcon, #353136, Corning Inc., Corning, NY, USA). The following day, non-adherent cells were collected and seeded at a cell density of 5.0 × 10^4^ cells/cm^2^ in a 48-well cell culture plate. Cells were treated with complete culture medium (see Supplementary Table S1) supplemented with 50 ng/mL M-CSF and RANKL, as well as CH digesta as listed in Table 1. CH digesta were dissolved in α-MEM with L-glutamine, without any additives, and filter-sterilized (0.22 µm) before cell culture application. RANKL was added at either 50 ng/mL (RANKL50) or 100 ng/mL (RANKL100), representing standard and enhanced osteoclastogenic stimulation, respectively. Cultures were maintained at 37 °C and 5% CO_2_ for 5–7 days with media changes on day 3 of culture and every other day afterward. Mature OCs were observed between days 5–7 and either fixed for tartrate-resistant acid phosphatase (TRAP) staining or collected for gene expression analysis. For each CH digesta, four doses (0.01, 0.05, 0.1, or 0.5 mg/mL) were assessed. Three types of controls were included in the study (Table 1): (1) a negative control comprising M-CSF treatment only, (2) a CH control comprising M-CSF and CH digesta without RANKL, and (3) a differentiation control with M-CSF and RANKL but no CH treatment.

### 2.4. Osteoclast Quantification and Analysis

At the end of the culture period, cell culture medium was removed, and mature OCs were fixed in 10% buffered formalin (pH 7.4) for 10 min at room temperature. TRAP staining was performed using a commercial kit (#387A-KT, Sigma-Aldrich, St. Louis, MO, USA), as described previously [30]. Images were obtained using a Cytation 5 Imaging Reader (Model CY5V, BioTek Instruments, Winooski, VT, USA) and processed using Gen5 Image Prime Software (Version 3.09.07, BioTek Instruments, Winooski, VT, USA). Mature OCs were defined as large, TRAP-positive cells with three or more nuclei. The number and area of OCs were determined using ImageJ software (Version 1.53) [31]. The area of each identified mature OC was measured individually, and the average area per mature OC was calculated for each analyzed culture.

### 2.5. Osteoblast Isolation and In Vitro Osteoblastogenesis

OBs were obtained from clean and bone marrow-flushed long bones of 11-week-old mice (nine males and three females), as described previously [32]. In brief, femora and tibiae were washed in 70% ethanol followed by 1X PBS (see Supplementary Table S1). Bones were placed in physiological solution (see Supplementary Table S1) and chopped into smaller pieces using sterile scissors. OBs were isolated by sequential enzymatic digestion using a three-step collagenase protocol [32]. Briefly, bone pieces were sequentially incubated with 1 mL of stock solutions 1, 2, and 3 (see Supplementary Table S1) for 15, 30, and 60 min, respectively. Stock solutions were changed between incubations. Afterwards, bone pieces were plated in a 10 cm Petri dish containing 10 mL of complete culture medium (see Supplementary Table S1). Cells were left to migrate out of the bone pieces over 5–10 days. Cultures were maintained at 37 °C with 5% CO_2_, and media were changed every other day until confluency was reached. Once confluent, cells were treated with 0.25% trypsin, passed through a 40 µm cell strainer (Fisherbrand, #22363547, Waltham, MA, USA), and resuspended in complete culture medium. Cells were plated onto 6-well plates at a density of 5000 cells/cm^2^ and left to acclimatize at 37 °C with 5% CO_2_ for 3 days. On day 3, media were changed, and 50 µg/mL ascorbic acid and 4 mM β-glycerophosphate were added alongside CH treatments, as described in Table 1. For the OB experiments, CH-GL and CH-OPT were evaluated at 0.01 and 0.1 mg/mL. The lower concentration was selected using the bioavailability-informed rationale described in Section 2.1.2, while 0.1 mg/mL provided a tenfold higher concentration within the range evaluated in the OC experiments. The OB arm was limited to these two concentrations because of the extended primary-cell culture period and the multiple gene-expression and staining endpoints. Cell culture media were changed every other day until day 28. At the end of the culture period, cells were either subjected to alizarin red, alkaline phosphatase, or Sirius red staining or collected for gene expression analysis. Treatments were compared to control conditions (Table 1).

### 2.6. Osteoblast Staining Analysis

OB monolayer cultures were fixed and subjected to alizarin red staining to assess mineralized bone nodules, alkaline phosphatase staining to assess osteoblastic differentiation, and Sirius red staining to evaluate collagen deposition. Staining was performed as previously described [32,33] and in accordance with the manufacturer’s instructions. Images were acquired using a Cytation 5 Imaging Reader (Model CY5V, BioTek Instruments, Winooski, VT, USA), visualized and processed using Gen5 Image Prime Software (Version 3.09.07, BioTek Instruments, Winooski, VT, USA) to quantify pixel intensity and stained area (µm^2^). Stained area was defined as the total stain-positive area per well, and pixel intensity was quantified separately as a measure of staining signal strength. Results were normalized to control conditions.

#### 2.6.1. Alizarin Red

OB monolayer cultures were rinsed with 1X PBS (see Supplementary Table S1) and fixed with 10% buffered formalin (pH 7.4) for 8–10 min at room temperature. Fixed OB monolayers were washed three times with 1X PBS and left to air dry. Once dry, wells were briefly rinsed with 70% ethanol and allowed to dry again. A 1% (*w*/*v*) alizarin red staining solution (#A5533, Sigma-Aldrich, St. Louis, MO, USA) was prepared in distilled water, filtered, and pH-adjusted to 5.5. The alizarin red staining solution was added to each cell layer, and the layers were incubated for 5–15 min at room temperature. Afterward, cells were washed three times with 50% ethanol, followed by distilled water and 1X PBS, before image acquisition.

#### 2.6.2. Alkaline Phosphatase

OB monolayer cultures were rinsed with 1X PBS (see Supplementary Table S1) and fixed with 10% buffered formalin (pH 7.4) for 8–10 min at room temperature. The staining solution was prepared by mixing Solution A—3.75 mL Milli-Q water, 0.2 M Tris-HCl (pH 8.3), and 4.5 mg Fast Red Violet salt (#F3381, Sigma-Aldrich, St. Louis, MO, USA)—with Solution B, which contained 0.75 mg naphthol (#N5000, Sigma-Aldrich, St. Louis, MO, USA) dissolved in 30 µL N,N-dimethylformamide (#BP1160, Fisher Scientific, Waltham, MA, USA). The final solution was filtered through a 0.22 µm membrane to remove precipitates. The staining solution was added to each cell layer and incubated for 8–15 min at room temperature in the dark. Afterward, cells were washed with distilled water until excess stain was removed and the wash solution ran clear. Images were then acquired.

#### 2.6.3. Sirius Red

OB monolayer cultures were rinsed with 1X PBS and fixed with ice-cold 70% ethanol for 1 h at 4 °C. Afterward, cells were washed with 1X PBS and left to air dry at room temperature. Once dry, cells were stained using the Picro-Sirius Red Stain Kit (#ab150681, Abcam, Cambridge, UK) according to the manufacturer’s instructions. Briefly, cells were incubated with Sirius red staining solution for 1 h with gentle agitation. Cells were then washed twice with the provided acetic acid solution, rinsed with double-distilled water, air-dried, and imaged.

### 2.7. Gene Expression Analysis

RNA was isolated from OC and OB cultures using TRIzol™ reagent (#15596026, Invitrogen, Thermo Fisher Scientific, Waltham, MA, USA) according to the manufacturer’s instructions. Reverse transcription was performed using the High-Capacity cDNA Reverse Transcription Kit (#4368813, Applied Biosystems, Thermo Fisher Scientific, Waltham, MA, USA), following the manufacturer’s instructions. Quantitative real-time PCR (qPCR) was performed using a QuantStudio™ 7 Flex System (Thermo Fisher Scientific, Software Version 1.3) with SYBR™ Green PCR Select Master Mix #4472918, Applied Biosystems, Thermo Fisher Scientific, Waltham, MA, USA) and customized primers (see Supplementary Table S2). An internal plate control was included on each PCR plate to enable comparisons across multiple PCR runs. *Beta-actin* and *GAPDH* were used as endogenous reference genes. For each target gene, ΔCt values were calculated relative to the endogenous reference genes, and relative expression was calculated using the ΔΔCt method [34], with values normalized to the respective control group.

### 2.8. Statistical Analysis

Statistical analyses and figure generation were performed using GraphPad Prism version 9.0.1 for Windows (GraphPad Software). The individual animal donor was considered the experimental unit, and technical replicates from the same donor were averaged before analysis. Gene-expression data were normalized to the respective control, log_2_-transformed, and analyzed using the transformed values. Phenotypic and staining outcomes are presented as fold change relative to the respective control, unless otherwise indicated. Normality was evaluated using formal normality testing and Q–Q plots, and homogeneity of variance was assessed using the Brown–Forsythe test. OC number and area values, which were normalized to a control value of 1.0, were analyzed using a two-tailed one-sample ratio *t*-test or Wilcoxon signed-rank test, as appropriate. Independent gene-expression datasets were analyzed using ordinary one-way ANOVA with Dunnett’s multiple-comparisons test, Welch’s ANOVA with Dunnett’s T3 test, or the Kruskal–Wallis test with Dunn’s test, as appropriate. Matched staining datasets were analyzed using repeated-measures one-way ANOVA with the Geisser–Greenhouse correction and Dunnett’s multiple-comparisons test. Data are reported as mean ± SEM. Statistical significance was defined as follows: * *p* < 0.05, ** *p* < 0.01, *** *p* < 0.001. All statistical comparisons were performed within each CH product separately, relative to its respective control. Treatment allocation, image acquisition and analysis, and statistical evaluation were not performed under blinded conditions.

## 3. Results

### 3.1. Effects of CH Digesta on Osteoclast Morphology and Gene Expression

**Figure 3:**
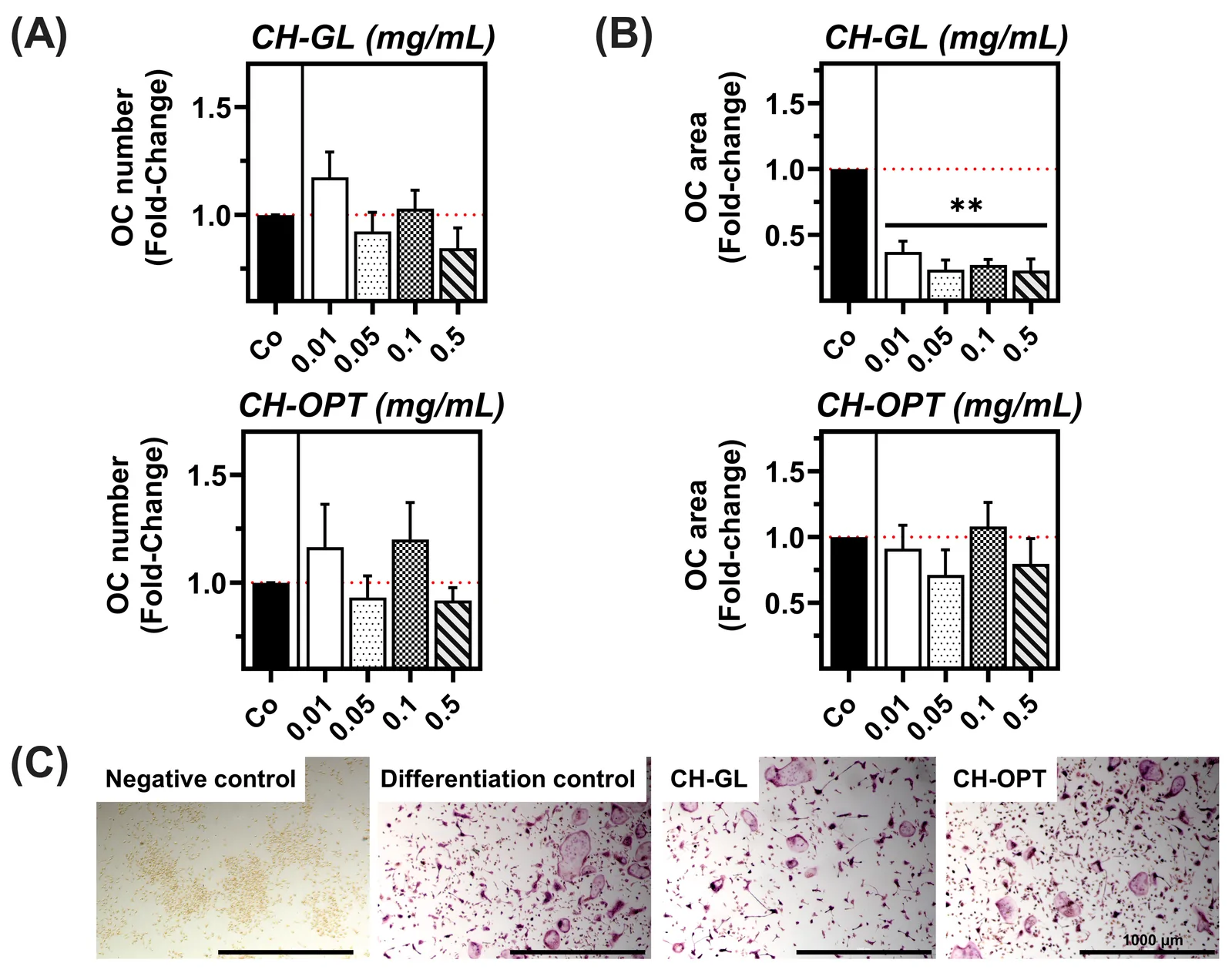
**Effects of CH treatment on OC number and area under standard RANKL conditions** (**50 ng/mL**)**.** Bone marrow-derived monocytes were differentiated into mature TRAP-positive OCs under standard osteoclastogenic conditions (M-CSF, 50 ng/mL; RANKL, 50 ng/mL) with or without CH digesta treatment. (**A**) Average number of differentiated OCs and (**B**) average area per mature OC (μm^2^) at different CH digesta doses, expressed as fold-change relative to the differentiation control (Co). (**C**) Representative images of TRAP-positive OCs (CH treatments shown at 0.5 mg/mL). The negative control represents cells treated with M-CSF (50 ng/mL) only, with no OC formation. Data are presented as mean ± standard error of the mean (SEM). Because OC number and area values were normalized to the corresponding differentiation control, which was set to 1.0 (red dotted line), each treatment group was compared with the hypothetical value of 1.0 using a two-tailed one-sample ratio *t*-test or a Wilcoxon signed-rank test, as appropriate. Statistical annotations indicate treatment-versus-control comparisons. For OC number, *n* = 8 biological replicates evaluated using three technical replicates per donor; for OC area, *n* = 5 biological replicates representing independent animal donors; ** *p* < 0.01.

We first confirmed that OC precursors did not form mature TRAP-positive OCs in the absence of RANKL (negative control; Figure 3C) or when exposed to CH digesta alone (CH controls; see Supplementary Figure S1), indicating that OC differentiation was only achieved via RANKL stimulation.

Next, CH-GL and CH-OPT were tested for effects on osteoclastogenesis under standard RANKL conditions. CH-GL did not significantly alter the number of TRAP-positive OCs (Figure 3A), but consistently reduced OC area by more than 50% across all tested doses (*p* < 0.01) (Figure 3B), as evident in representative TRAP-stained images (Figure 3C). Thus, the apparent reduction in cellular coverage in the representative images is consistent with the marked reduction in average OC area rather than a decrease in the quantified number of mature TRAP-positive OCs.

**Figure 4:**
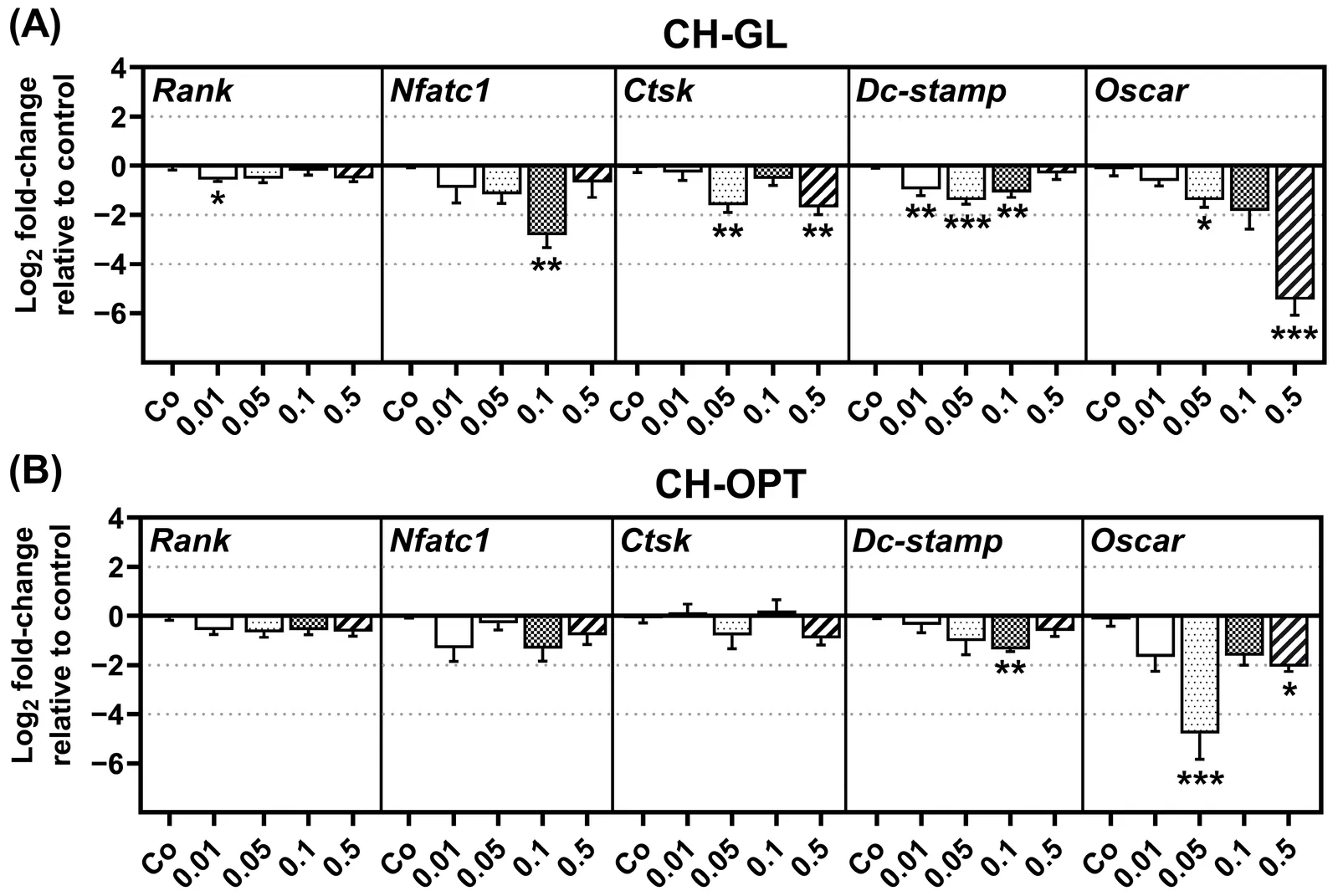
**CH digesta modulate osteoclastogenic gene expression under standard RANKL conditions.** Gene expression levels were assessed in OCs treated with (**A**) CH-GL or (**B**) CH-OPT (0.01, 0.05, 0.1, or 0.5 mg/mL) under standard RANKL differentiation conditions. Data are presented as mean ± SEM and expressed as log_2_-transformed fold-change relative to the differentiation control (Co). Statistical significance was determined using ordinary one-way ANOVA followed by Dunnett’s multiple-comparisons test, Welch’s ANOVA followed by Dunnett’s T3 multiple-comparisons test, or the Kruskal–Wallis test followed by Dunn’s multiple-comparisons test, as appropriate. Statistical annotations indicate treatment-versus-control comparisons. The analysis included *n* = 5–8 biological replicates per group, evaluated using two technical replicates per donor; * *p* < 0.05, ** *p* < 0.01, *** *p* < 0.001.

To explore transcriptional changes associated with this phenotype, we assessed *Rank* and selected downstream osteoclastogenic genes. In the presence of 0.01 mg/mL CH-GL, *Rank* expression was significantly reduced (*p* < 0.05) (Figure 4A). Nuclear factor of activated T cells 1 (*Nfatc1*), a major transcriptional regulator downstream of RANK signaling, was significantly downregulated at 0.1 mg/mL CH-GL (*p* < 0.01) (Figure 4A). The cathepsin K gene (*Ctsk*), which encodes a major enzyme involved in type I collagen degradation [35], showed significantly reduced expression at 0.05 and 0.5 mg/mL CH-GL (*p* < 0.01) (Figure 4A). *Dendritic cell-specific transmembrane protein* (*Dc-stamp*), a key regulator of pre-osteoclast cell fusion [36], was significantly decreased at 0.01 (*p* < 0.01) and at 0.05 and 0.1 mg/mL (*p* < 0.001) (Figure 4A). In addition to the RANK/RANKL pathway, we also investigated the co-stimulatory pathway via osteoclast-associated receptor (OSCAR), which is important for complete osteoclastic differentiation [37,38]. Mean *Oscar* expression showed an overall downward pattern across the tested CH-GL concentrations, with significant reductions at 0.05 mg/mL (*p* < 0.05) and 0.5 mg/mL (*p* < 0.001) (Figure 4A). Because *Dc-stamp* and *Oscar* support pre-OC fusion and full differentiation, these data align with the reduction in OC area and suggest that altered cell–cell fusion may contribute to this change.

For CH-OPT, OC number and OC area were not significantly altered under standard RANKL conditions (Figure 3A,B). Gene expression was largely unchanged for *Rank*, *Nfatc1*, and *Ctsk* (Figure 4B), although *Dc-stamp* was reduced at 0.1 mg/mL (*p* < 0.01). *Oscar* expression was significantly reduced at 0.05 mg/mL (*p* < 0.001) and 0.5 mg/mL (*p* < 0.05) (Figure 4B). The reductions were not significant at 0.01 or 0.1 mg/mL, indicating a concentration-specific response. The absence of corresponding morphological changes suggests that, despite these selective transcriptional changes, CH-OPT was insufficient to modify overt OC differentiation outcomes under these conditions.

To assess responses under stronger osteoclastogenic stimulation, we also explored the effects of CH-GL and CH-OPT on osteoclastogenesis under high RANKL conditions (100 ng/mL) (Supplementary Figure S2). CH-GL maintained its inhibitory effect on OC area across all doses (*p* < 0.01) (Supplementary Figure S2B), without significantly affecting OC number (Supplementary Figure S2A). Under these conditions, CH-GL reduced *Rank* expression at all tested doses and decreased *Ctsk* expression at 0.01 mg/mL (Supplementary Figure S3A). *Nfatc1* and *Dc-stamp* expression were not significantly altered, whereas *Oscar* expression was significantly increased at 0.5 mg/mL. Thus, under stronger RANKL stimulation, CH-GL continued to reduce OC area and alter OC morphology, with a more selective transcriptional footprint.

Under high RANKL conditions, CH-OPT again did not significantly affect OC number or area (Supplementary Figure S2A,B). However, CH-OPT reduced *Rank* expression at 0.01, 0.05, and 0.1 mg/mL, decreased *Ctsk* expression at 0.05 mg/mL, and lowered *Nfatc1* at 0.1 mg/mL (Supplementary Figure S3B). *Dc-stamp* expression was unchanged, while *Oscar* expression was significantly increased at 0.1 mg/mL (Supplementary Figure S3B).

Finally, we further evaluated leukocyte-associated immunoglobulin-like receptor 1 (*Lair-1*), an inhibitory receptor on pre-OCs that binds collagen fragments and dampens osteoclastogenesis [39]. Under high RANKL conditions, CH-GL significantly reduced *Lair-1* expression at 0.05 and 0.5 mg/mL. *Lair-1* expression was not significantly altered following CH-OPT treatment at any tested concentration (Supplementary Figure S3A,B). The CH-GL-associated decrease may indicate altered regulation of a collagen-sensitive inhibitory pathway during strong RANKL stimulation.

Together, these results show that CH-GL selectively restricts osteoclast size, possibly through effects on pre-OC fusion and maturation, without reducing cell number, and this effect persists even under high RANKL. For CH-OPT, selected context-dependent transcriptional changes were observed, while OC number and area were not significantly altered under the conditions tested.

### 3.2. Effects of CH Digesta on Osteoblast Gene Expression and Matrix-Associated Outcomes

**Figure 5:**
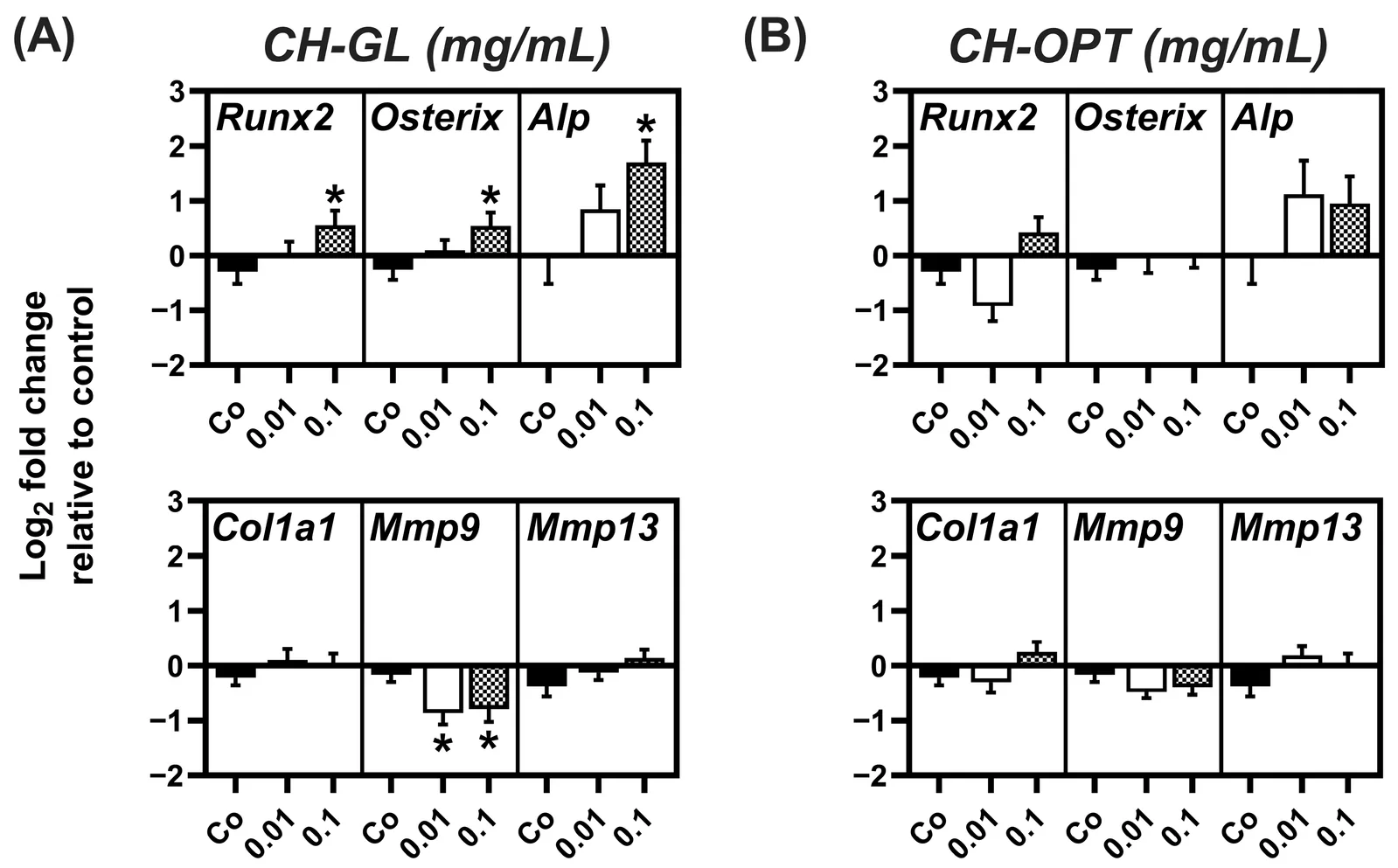
**CH digesta treatment modulates osteoblastic gene expression.** Primary OBs were cultured in osteogenic medium containing β-glycerophosphate and ascorbic acid. Cells were either untreated (control) or treated with 0.01 or 0.1 mg/mL of (**A**) CH-GL or (**B**) CH-OPT digesta. Gene expression levels of *Runx2*, *Osterix*, *Alp*, *Col1a1*, *Mmp9*, and *Mmp13* were determined and expressed as log_2_-transformed fold-change relative to the untreated control (Co). Data are reported as mean ± SEM. Statistical significance was assessed using one-way ANOVA with Dunnett’s post hoc test or Kruskal–Wallis test followed by Dunn’s multiple-comparisons test comparing treatment doses with their respective control, as appropriate (*n* = 8–12 biological replicates per group, evaluated using two technical replicates per donor; * *p* < 0.05).

Next, we examined OB differentiation by assessing key osteoblast lineage regulators, including *Runx2* and *Osterix* [38,40,41], after exposure to CH digesta. Compared to the control, CH-GL selectively enhanced osteogenic gene expression at 0.1 mg/mL, increasing *Runx2*, *Osterix*, and *Alp* (*p* < 0.05), while collagen type I (*Col1a1*) was unchanged (Figure 5A). We further evaluated the expression of matrix metalloproteinases involved in bone matrix remodeling. CH-GL concurrently reduced matrix metalloproteinase 9 (*Mmp9*) at 0.01 and 0.1 mg/mL (*p* < 0.05) without affecting *Mmp13* (Figure 5A), suggesting a selective reduction in *Mmp9* expression rather than a broad suppression of matrix metalloproteinase-related genes.

**Figure 6:**
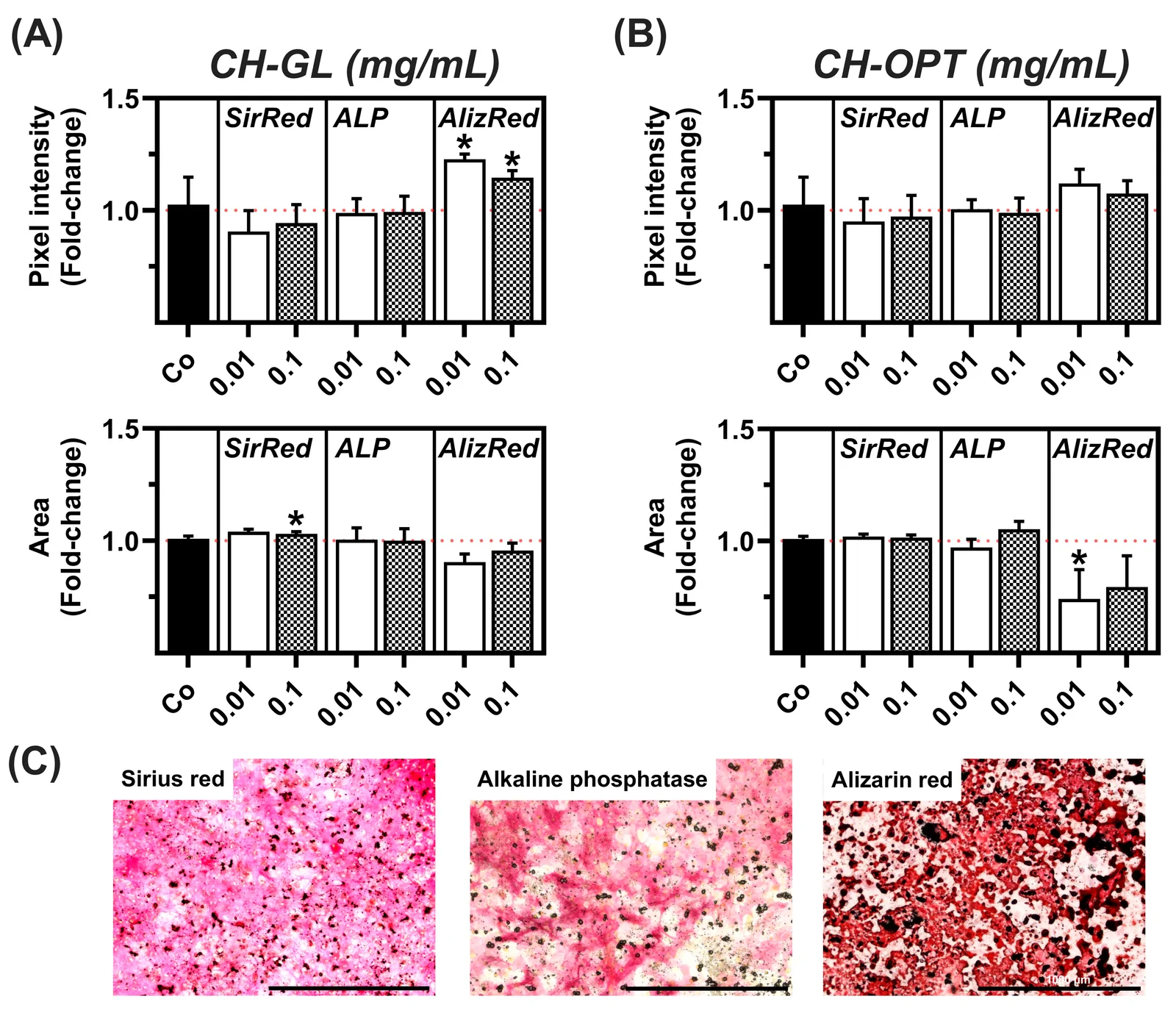
**CH digesta treatment affects osteoblastic matrix staining outcomes.** Primary OBs were cultured for a total of 28 days. Following a 3-day acclimatization period, osteogenic supplements and CH treatments were initiated on day 3 and maintained until collection on day 28. (**A**,**B**) Quantitative analysis of Sirius red (SirRed), alkaline phosphatase (ALP), and Alizarin red (AlizRed) staining following (**A**) CH-GL and (**B**) CH-OPT treatment. Results are presented as pixel intensity and total stain-positive area per well (μm^2^), expressed as fold-change relative to the differentiation control (Co), which was set to 1.0 (red dotted line). (**C**) Representative images of osteogenic differentiation control cultures without CH digesta treatment (scale bar = 1000 μm). Data are reported as mean ± SEM. For each staining outcome and CH product, treatment groups were compared with the corresponding differentiation control using repeated-measures one-way ANOVA with the Geisser–Greenhouse correction, followed by Dunnett’s multiple-comparisons test. Statistical annotations indicate treatment-versus-control comparisons. The analysis included *n* = 10–11 biological replicates for Sirius red and ALP and *n* = 5–6 biological replicates for Alizarin red, representing independent animal donors; * *p* < 0.05.

Evaluation of collagen deposition (Sirius red), alkaline phosphatase (ALP) staining, and calcium deposition (Alizarin red) partially aligned with a modest pro-osteogenic profile of CH-GL. We assessed both the total stained area and pixel intensity, with the latter serving as a proxy for signal strength and overall stain density. CH-GL produced a small but significant increase in Sirius red–positive area at 0.1 mg/mL (*p* < 0.05), with no change in staining intensity, suggesting slightly greater collagen-associated coverage rather than denser collagen deposition (Figure 6A). ALP histochemistry was unchanged (Figure 6A), while Alizarin red intensity was increased at 0.01 and 0.1 mg/mL (*p* < 0.05), consistent with enhanced mineralization-related staining intensity (Figure 6A); however, the mineralized area itself did not expand. Together, these data suggest that CH-GL modestly supports selected osteoblast differentiation-related markers and matrix/mineralization-associated staining outcomes, without broadly increasing all measured osteogenic endpoints.

For CH-OPT, no significant changes were observed in *Runx2*, *Osterix*, *Alp*, *Col1a1*, *Mmp9*, or *Mmp13* expression at the concentrations tested (Figure 5B). Collagen deposition and ALP staining were also unchanged (Figure 6B). Alizarin red intensity was not significantly altered, while the mineralized area was significantly reduced at 0.01 mg/mL (*p* < 0.05), suggesting a possible reduction in mineralized matrix coverage at the low concentration.

## 4. Discussion

This study addresses a major research gap by examining the direct effects of CH digesta on primary murine bone remodeling cells. Overall, the findings identified changes in selected OC- and OB-related outcomes following CH digesta treatment. In OC cultures, CH-GL markedly reduced OC area without significantly altering OC number under both standard and high RANKL conditions (Figure 3; Supplementary Figure S2). Consistent with this morphological change, CH-GL downregulated selected osteoclastogenic, fusion-related, and resorption-associated genes, including *Nfatc1*, *Ctsk*, *Dc-stamp*, and *Oscar* (Figure 4). In OB cultures, CH-GL modestly enhanced selected osteogenic markers, including *Runx2*, *Osterix*, and *Alp*, while reducing *Mmp9* expression and producing limited but supportive changes in mineralization- and collagen-associated staining outcomes (Figure 5 and Figure 6). For CH-OPT, selected changes were observed in OC-related gene expression, while OC area and most assessed OB-related outcomes were not significantly altered. Because no direct statistical comparisons were performed between CH-GL and CH-OPT, apparent differences in their response patterns should be interpreted descriptively rather than as evidence of statistically significant between-product differences.

The distinction between OC number and OC area is an important aspect of the present findings. Under both standard and high RANKL conditions, CH-GL did not significantly reduce the number of differentiated OCs, but consistently and markedly reduced OC area (Figure 3; Supplementary Figure S2). OC number reflects how many precursor cells reached the TRAP-positive OC stage, whereas OC area reflects the size, spreading, and morphological maturity of the OCs that formed. Thus, CH-GL did not appear to suppress OC differentiation at the level of OC number, but rather limited the development of larger OC-like cells. This distinction is biologically critical because OC size is positively correlated with resorptive capacity; larger, highly multinucleated OCs exhibit significantly greater bone-resorbing activity compared to smaller variants [42]. Therefore, the observed reduction in OC area may reflect altered morphology among the OCs that formed, potentially involving reduced cell spreading and/or reduced fusion into larger multinucleated osteoclasts [36,43,44]. Consistent with this interpretation, the unchanged number of mature OCs did not suggest a substantial loss of mature OCs under the conditions tested. Future studies incorporating dedicated viability and apoptosis assays would directly assess these outcomes. However, because direct functional resorption assays were not performed, these findings highlight altered OC morphology rather than providing definitive proof of impaired bone resorptive activity. Importantly, this focus on morphological parameters expands upon previous collagen-related research, which has primarily quantified OC number or general differentiation status without fully considering changes in OC area or morphology [6,7,11].

The gene expression profile under standard RANKL conditions further supports the interpretation that CH-GL affected specific later-stage features of OC development rather than preventing OC formation entirely. Although *Rank* expression was largely unchanged except at the lowest CH-GL dose, CH-GL downregulated several downstream osteoclastogenic markers, including *Nfatc1*, *Ctsk*, *Dc-stamp*, and *Oscar* (Figure 4A). *Nfatc1* is a central transcriptional regulator of osteoclastogenesis, and *Ctsk*, one of its established downstream targets, encodes a major protease involved in type I collagen degradation [45]. However, the significant changes in these genes occurred at different CH-GL concentrations. Thus, the reduced *Nfatc1* expression observed at 0.1 mg/mL CH-GL may indicate partial suppression of the transcriptional program associated with OC maturation, whereas the reductions in *Ctsk* expression at 0.05 and 0.5 mg/mL should be interpreted as separate dose-specific responses rather than as evidence that the *Ctsk* changes were directly mediated by *Nfatc1* in the present study. The reduction in *Ctsk* expression is also consistent with a previous study using RAW264.7 OC precursor cells, in which undigested chicken collagen extract reduced *Ctsk* expression [7]. In parallel, CH-GL significantly downregulated *Dc-stamp* at selected doses (Figure 4A). As *Dc-stamp* is involved in pre-osteoclast fusion [36], its downregulation provides a plausible explanation for the reduced OC area: OCs may still form, but with reduced fusion or expansion into larger OC-like cells. This interpretation is further supported by previous CH-related work using an ex vivo serum-based model, in which bone marrow cells were exposed to serum collected after oral hydrolyzed collagen intake, resulting in reduced expression of CD36, another fusion-associated marker [6]. Given the established role of *Dc-stamp* in pre-osteoclast fusion, its modulation by CH-GL further supports the possibility that CH digesta may affect fusion-related aspects of later-stage osteoclastogenesis. *Oscar* expression was also significantly reduced by CH-GL at 0.05 and 0.5 mg/mL (Figure 4A). OSCAR is an FcRγ-associated collagen receptor whose engagement by specific structural motifs in fibrillar collagen provides costimulatory signaling during osteoclastogenesis [46,47]. Because the treatments consisted of collagen-derived digesta, the reductions in *Oscar* mRNA observed at selected CH-GL concentrations may reflect altered regulation of this collagen-responsive pathway. However, the present study did not assess whether the digesta contained structurally compatible OSCAR ligands or directly engaged OSCAR; therefore, the observed mRNA changes do not establish altered receptor binding or signaling. Together, these transcriptional changes suggest that CH-GL partially dampened the expression of selected genes related to OC fusion, maturation, and resorption-associated function, including collagen matrix degradation, while the unchanged OC number indicates that CH-GL did not fully suppress the formation of TRAP-positive OCs. Future studies should investigate whether CH-derived collagen fragments influence additional regulatory pathways that act in parallel with RANK/RANKL signaling [39,48]. To further clarify the underlying mechanisms, time-course protein-level analyses should assess whether CH digesta modulate early RANKL-induced signaling, including activation of the canonical and non-canonical NF-κB pathways. To our knowledge, this is among the first studies to report modulation of *Dc-stamp* and *Oscar* expression following CH digesta treatment.

Beyond their roles in osteoclastogenesis, DC-STAMP and OSCAR also contribute to dendritic-cell biology. DC-STAMP has been implicated in the regulation of dendritic-cell antigen presentation and immune tolerance, whereas collagen-mediated OSCAR engagement can promote the activation and maturation of human monocyte-derived dendritic cells and stimulate cytokine production [49,50]. These shared functions highlight an interface between bone remodeling and immune regulation and suggest that the observed changes in *Dc-stamp* and *Oscar* expression may have broader osteoimmune relevance. Although these genes were assessed only in OC cultures, the findings provide a rationale for future studies examining whether CH digesta also influence dendritic-cell maturation, antigen presentation, or cytokine responses.

Under high RANKL conditions, the response to CH digesta appeared more complex at the transcriptional level. Morphologically, CH-GL maintained its effect on OC area without significantly altering OC number (Supplementary Figure S2). CH-OPT did not significantly affect either morphological endpoint. At the transcriptional level, both CH-GL and CH-OPT reduced *Rank* expression across multiple doses. CH-GL additionally reduced *Ctsk* and *Lair-1* expression at selected doses and increased *Oscar* expression at 0.5 mg/mL. CH-OPT reduced *Nfatc1* and *Ctsk* expression at selected doses and increased *Oscar* expression at 0.1 mg/mL. *Dc-stamp* expression was unchanged following either treatment, while *Lair-1* expression was unchanged following CH-OPT treatment (Supplementary Figure S3). These findings suggest that stronger RANKL stimulation may partly uncouple the transcriptional and morphological responses of OCs to CH digesta, possibly due to saturation of osteoclastogenic differentiation pathways. Since these gene expression changes did not consistently translate into changes in OC number or area, the high RANKL data should be interpreted cautiously as exploratory rather than as a direct extension of the standard RANKL mechanism. To clarify these findings, future studies should examine OC responses across graded RANKL concentrations using direct resorption assays and protein-level signaling analyses.

In OB cultures, CH-GL treatment was associated with changes in selected osteogenic markers. Consistent with previous reports that collagen-derived peptides can upregulate OB differentiation markers [20,51], 0.1 mg/mL CH-GL increased the expression of *Runx2* and *Osterix* (Figure 5A), two key transcriptional regulators involved in OB differentiation and bone formation [52,53]. CH-GL also increased *Alp* expression at 0.1 mg/mL, suggesting a modest shift toward an osteogenic transcriptional profile (Figure 5A). CH-OPT did not significantly alter the assessed OB differentiation markers relative to its control (Figure 5B). Beyond osteogenic markers, CH-GL significantly reduced *Mmp9* expression at both tested doses (Figure 5A). *Mmp9* expression was not significantly altered following CH-OPT treatment (Figure 5B). Given that elevated MMP-9 has been implicated in OA-associated matrix degradation [54], reduced *Mmp9* expression may suggest a lower matrix-degrading gene profile following CH-GL treatment. The effects of CHs on MMP-related responses in bone cells remain poorly characterized [55,56]. However, because MMP activity was not directly assessed, this interpretation should be limited to the gene expression level. In contrast, *Mmp13*, a key enzyme in cartilage degradation [57], remained unchanged across treatments (Figure 5A,B), indicating that CH digesta did not broadly suppress matrix metalloproteinase-related genes under these conditions. Although *Osterix* has been reported to activate the *Mmp13* promoter in a dose-dependent manner [52], the CH-GL-induced increase in *Osterix* expression did not translate into altered *Mmp13* expression in the present study.

The functional staining outcomes partially supported the OB gene expression findings, although the relationship was not fully concordant. CH-GL produced modest increases in mineralization-related signal intensity, as reflected by increased Alizarin red pixel intensity, and a small increase in collagen-associated stained area, as reflected by Sirius red staining (Figure 6A). These findings are consistent with modest support for matrix/mineralization-related outcomes, but they should not be interpreted as broad enhancement of OB function. For example, although *Alp* gene expression was increased by CH-GL (Figure 5A), ALP staining intensity and stained area were not significantly altered (Figure 6A). Similarly, *Col1a1* expression was not significantly changed (Figure 5A,B), despite the small increase in Sirius red-stained area following CH-GL treatment (Figure 6A). Together, these findings suggest that transcriptional changes did not fully translate into corresponding staining-level outcomes, likely because mRNA expression, enzyme activity, matrix accumulation, and mineral deposition represent different stages of the osteogenic process and may not change in parallel [58]. For CH-OPT, no significant increases were observed in the assessed OB gene-expression or mineralization-related staining outcomes (Figure 5B and Figure 6B), while Alizarin red-stained area was reduced at 0.01 mg/mL (Figure 6B). Collectively, the within-product findings indicate that CH-GL treatment was associated with modest changes in selected OB osteogenic markers and matrix/mineralization-related staining outcomes.

Previous characterization of CH-GL and CH-OPT identified differences in their digestion-derived peptide profiles and estimated peptide bioavailability [22,23]. These included higher glycyl-prolyl-hydroxyproline (Gly-Pro-Hyp) and Pro-Hyp-Gly content in CH-GL, several unidentified peptide peaks unique to each product, and transport of Gly-Pro-Hyp following CH-GL treatment in the HIEC-6/HepG2 model [22,23]. These compositional observations provide hypotheses for future mechanistic investigation but cannot presently be linked to the within-product cellular responses observed in this study.

The actual Pro-Hyp and total peptide concentrations were not measured in the specific treatment preparations, and the digesta consisted of complex mixtures containing peptides, free amino acids, salts, and other low-molecular-weight components. Therefore, the present study could not determine the specific component(s) responsible for the observed biological responses. Targeted fractionation with bioactivity-guided testing of purified fractions and individual candidate components will be required to address this question.

An important methodological strength of the present study is the use of CH digesta rather than intact CHs applied directly to bone remodeling cells. Previous in vitro studies have often used intact CHs, thereby overlooking the effects of GI digestion and peptide bioavailability [4,59]. Although one previous study addressed this limitation by applying human serum collected after oral CH intake to bone-related cell cultures [6], direct measurement of BAPs in the tested serum was not performed, and serum-based approaches can be limited by practical and ethical constraints. In this context, the combination of in vitro digestion, bioavailability-informed CH digesta preparation, and primary bone cell assays provides a feasible approach to evaluate bone-cell responses to digestion-derived CH fractions [22,23]. However, the present model used primary bone cells from young, healthy mice and did not reproduce oral absorption, systemic metabolism, or the multicellular, inflammatory, and biomechanical OA joint environment. Accordingly, the relevance of these findings to OA is preliminary and indirect. A further limitation is that neither a collagen-free enzyme-only digestion control nor a matched non-collagen protein digest was included. Consequently, it remains unclear whether the observed responses were specific to collagen-derived material, would also occur with other protein digests, or were influenced by digestion-related background components. Although 10 kDa ultrafiltration is expected to reduce the presence of intact digestive enzymes, the potential carryover of low-molecular-weight components from the enzyme preparations cannot be entirely ruled out. Future studies should include both controls. Other limitations include the need for direct resorption assays to confirm whether the reduced OC area observed with CH-GL translates into altered OC function, and for targeted peptide fractionation to identify the digesta components that may have contributed to the observed cellular responses. Finally, in vivo studies will also be needed to determine whether these cell-based responses translate into measurable changes in bone remodeling within the joint environment.

## 5. Conclusions

This study demonstrates that CH digesta modulated selected outcomes in primary murine bone remodeling cells. CH-GL digesta consistently reduced OC area without significantly altering OC number, suggesting an effect on OC morphology rather than complete suppression of OC formation. This was accompanied by reduced expression of selected osteoclastogenic, fusion-related, and resorption-associated genes. In OB cultures, CH-GL modestly increased selected osteogenic markers and mineralization-related staining intensity while reducing *Mmp9* expression. For CH-OPT, selected changes in OC-related gene expression were observed, while OC morphology and most assessed OB-related outcomes were not significantly altered under the tested conditions. Overall, further functional assays, peptide fractionation, and in vivo studies are warranted to clarify the mechanisms and physiological relevance of the observed cellular responses.

## Figures and Tables

**Table 1:** Applied cell culture conditions. Description of controls and treatments.

Osteoclastogenesis	
***Treatment***	*Media supplements*
**Negative Control**	M-CSF
**CH-GL CH control**	M-CSF + CH-GL (0.01 and 0.1 mg/mL)
**CH-OPT CH control**	M-CSF + CH-OPT (0.01 and 0.1 mg/mL)
**Differentiation Control 1** (**RANKL50**)	M-CSF + RANKL50
**Differentiation Control 2** (**RANKL100**)	M-CSF + RANKL100
**CH-GL**	M-CSF + RANKL50 + CH-GL (0.01, 0.05, 0.1, 0.5 mg/mL)
	M-CSF + RANKL100 + CH-GL (0.01, 0.05, 0.1, 0.5 mg/mL)
**CH-OPT**	M-CSF + RANKL50 + CH-OPT (0.01, 0.05, 0.1, 0.5 mg/mL)
	M-CSF + RANKL100 + CH-OPT (0.01, 0.05, 0.1, 0.5 mg/mL)
**Osteoblastogenesis**	
***Treatment***	*Media supplements*
**Control**	Ascorbic acid + β-glycerophosphate
**CH-GL**	Ascorbic acid + β-glycerophosphate + CH-GL (0.01 mg/mL)
	Ascorbic acid + β-glycerophosphate + CH-GL (0.1 mg/mL)
**CH-OPT**	Ascorbic acid + β-glycerophosphate + CH-OPT (0.01 mg/mL)
	Ascorbic acid + β-glycerophosphate + CH-OPT (0.1 mg/mL)

## Data Availability

The original contributions presented in this study are included in the article/Supplementary Material. Further inquiries can be directed to the corresponding author.

## Supplementary Materials

The following supporting information can be downloaded at: https://www.mdpi.com/article/10.3390/nu18162688/s1, Table S1: Cell culture media and solutions; Table S2: Primer list for qPCR analysis; Table S3: *p* values for comparisons of gene expression between the two RANKL controls (50 and 100 ng/mL); Figure S1: OC differentiation was not induced by CH treatment alone; Figure S2: Exploratory analysis of the effects of CH digesta treatment on OC number and area under enhanced osteoclastogenic stimulation (RANKL, 100 ng/mL); Figure S3: Exploratory analysis of CH digesta effects on osteoclastogenic gene expression under enhanced osteoclastogenic stimulation (RANKL, 100 ng/mL).

## Abbreviations

The following abbreviations are used in this manuscript:

ALP	Alkaline phosphatase
ANOVA	Analysis of variance
BAPs	Bioactive peptides
CH	Collagen hydrolysate
CH-GL	Collagen hydrolysate—Genacol Original Formula ^®^
CH-OPT	Collagen hydrolysate—Selection
Col1a1	Collagen type I alpha 1 chain
Ctsk	Cathepsin K
Dc-stamp	Dendritic cell-specific transmembrane protein
GAPDH	Glyceraldehyde 3-phosphate dehydrogenase
GI	Gastrointestinal
Lair-1	Leukocyte-associated immunoglobulin-like receptor 1
M-CSF	Macrophage colony-stimulating factor
MMP	Matrix metalloproteinase
Nfatc1	Nuclear factor of activated T cells 1
OA	Osteoarthritis
OB	Osteoblast
OC	Osteoclast
Oscar	Osteoclast-associated receptor
Osterix	Sp7 transcription factor
Pro-Hyp	Prolyl-hydroxyproline
qPCR	Quantitative polymerase chain reaction
RANK	Receptor activator of nuclear factor kappa-B
RANKL	Receptor activator of nuclear factor kappa-B ligand
Runx2	Runt-related transcription factor 2
SEM	Standard error of the mean
TRAP	Tartrate-resistant acid phosphatase
