# Supplementary material for: Differential Effects of Collagen Hydrolysate Digesta on Osteoclast and Osteoblast Responses In Vitro
Source: Nutrients. 2026 Aug 18;18(16):2688. doi: 10.3390/nu18162688 (PMC13516267; doi:10.3390/nu18162688)
Supplement: Supplementary file 1 [file nutrients-18-02688-s001.zip › nutrients-4414702-supplementary.pdf]

Differential Effects of Collagen Hydrolysate Digesta on Osteoclast and Osteoblast Responses In Vitro

Sirui Shan[1], Christina E. Larder [1,2], Josephine T. Tauer [3,4,5], Michèle M. Iskandar [1], Svetlana V. Komarova [3,4,6], and Stan Kubow [1*]

[1] School of Human Nutrition, McGill University, Ste-Anne-de-Bellevue, QC, Canada, H9X 3V9
[2] Corporation Genacol Canada Inc., Blainville, QC, Canada, J7C 6B4
[3] Faculty of Dental Medicine and Oral Health Sciences, McGill University, Montréal, QC, Canada, H3A 1G1
[4] Shriners Hospitals for Children-Canada, Montréal, QC, Canada, H4A 0A9
[5] Department of Pediatrics and Adolescent Medicine, Johannes Kepler University, Linz, Austria, 4020
[6] Department of Biomedical Engineering, Faculty of Engineering, University of Alberta, Edmonton, Alberta, Canada, T6G 1H9
* Correspondence: stan.kubow@mcgill.ca; Tel.: +1-514-398-7754

Supplementary Materials

Table S1. Cell culture media and solutions

	**Components**
**Base media**	αMEM with L-glutamine (Gibco, #12000-022)
**Complete media**	Base media, 10% fetal bovine serum (FBS, Wisent, #080152), 1% sodium pyruvate (Wisent, #600-110-EL), 1% penicillin-streptomycin (Wisent, #450-201-EL), 0.1 mg/mL ampicillin (BioShop Canada Inc., #AMP201.25)
**1x PBS**	140 mM NaCl, 3 mM KCl, 10 mM Na_2HPO_4, 2 mM KH_2PO_4, 500 mL MilliQ H_2O, adjusted to pH 7.4
**Physiological solution**	10 mM glucose, 130 mM NaCl, 5 mM KCl, 1 mM $MgCl_2$, 1 mM $CaCl_2$, 20 mM HEPES
**Stock solution 1**	10 mL physiological solution, 125 µL 0.25% trypsin, 5 µL collagenase P (100 mg/mL) (Thermo Fisher, #11213857001)
**Stock solution 2**	10 mL physiological solution, 125 µL 0.25% trypsin, 10 µL collagenase P (100 mg/mL) (Thermo Fisher, #11213857001)
**Stock solution 3**	10 mL physiological solution, 125 µL 0.25% trypsin, 100 µL collagenase II (100 mg/mL) (Worthington Biochemical, #CollagenaseII)

Table S2. Primer list for qPCR analysis

Gene	Sequence 5' - 3'		NCBI Reference Sequence
**Osteoclasts**			
Rank	Forward	GCATCCCTTGCAGCTCAACA	NM_009399.4
	Reverse	ATGGAAGAGCTGCAGACCAC	
Oscar	Forward	TCGCTGATACTCCAGCTGTC	NM_175632.3
	Reverse	TCTGGGGAGCTGATCCGTTA	
Cathepsin K	Forward	CAGTAGCCACGCTTCCTATCC	NM_007802.4
	Reverse	ACGCCGAGAGATTTCATCCA	
Lair-1	Forward	CTGTACCCCTGGGCAACTTT	NM_001302681.1
	Reverse	TTCCATAAAGGTGCTGCCGT	
Nfatc1	Forward	CCCGGAGTTCGACTTCGATT	NM_016791.4
	Reverse	TCTCTGTAGGCTTCCAGGCT	
Dc-stamp	Forward	TTTCCACGAAGCCCTAGCTG	NM_029422.4
	Reverse	GCGTTCCTACCTTCACGGAG	
**Osteoblasts**			
Col1a1	Forward	GAGCGGAGAGTACTGGATCG	NM_007742.4
	Reverse	GTTCGGGCTGATGTACCAGT	
ALP	Forward	CAGGCCGCCTTCATAAGCA	NM_007431.3
	Reverse	GTGCCGATGGCCAGTACTAA	
Runx2	Forward	GCTTCTCAGCTTTAGCGTCG	NM_001145920.2
	Reverse	AAGGTGCCGGGAGGTAAGT	
Osterix	Forward	GATGGCGTCCTCTCTGCTTG	NM_130458.4
	Reverse	GGGCTGAAAGGTCAGCGTAT	
MMP9	Forward	CCAGCCGACTTTTGTGGTCT	NM_013599.4
	Reverse	TGGCCTTTAGTGTCTGGCTG	
MMP13	Forward	GCCATTACCAGTCTCCGAGG	NM_008607.2
	Reverse	GGTCACGGGATGGATGTTCA	
**Housekeeping Genes (endogenous controls)**			
β-Actin	Forward	TGTTACCAACTGGGACGACA	NM_007393.5
	Reverse	GGGGTGTTGAAGGTCTCAAA	
GAPDH	Forward	ACCCAGAAGACTGTGGATGG	NM_001289726.1
	Reverse	CACATTGGGGGTAGGAACAC	

Table S3. *P* values for comparisons of gene expression between the two RANKL controls (50 and 100 ng/mL).

Gene	*p* value
Rank	0.8735
Lair-1	0.9998
Oscar	0.9993
Nfatc1	0.9995
Dc-stamp	0.9977
Cathepsin K	0.9446

For each gene, statistical significance was assessed by a *t*-test ($p < 0.05$) to determine differences between RANKL controls (50 ng/mL versus 100 ng/mL).

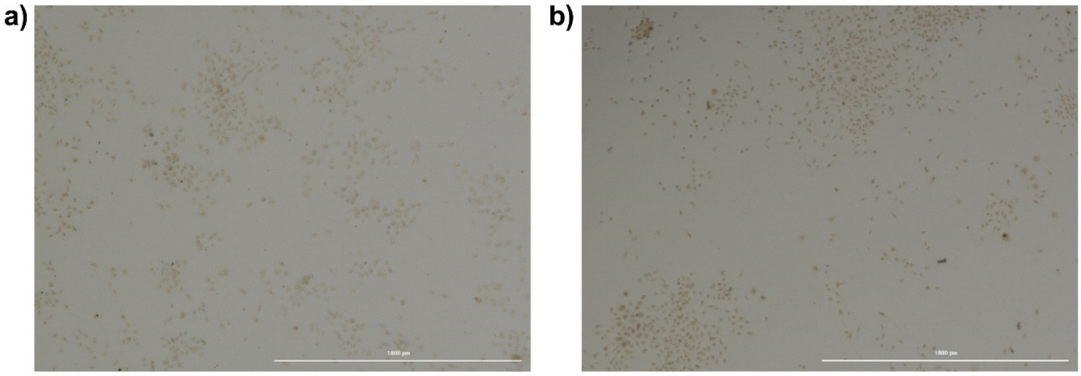

Figure S1. OC differentiation was not induced by CH treatment alone. Representative images of TRAP-stained OC precursor cells plated with M-CSF (50 ng/mL) and CH digesta (0.5 mg/mL) with no RANKL. No wells showed positively stained OCs after treatment with either: (a) CH-GL or (b) CH-OPT (scale bar = 1000 µm).

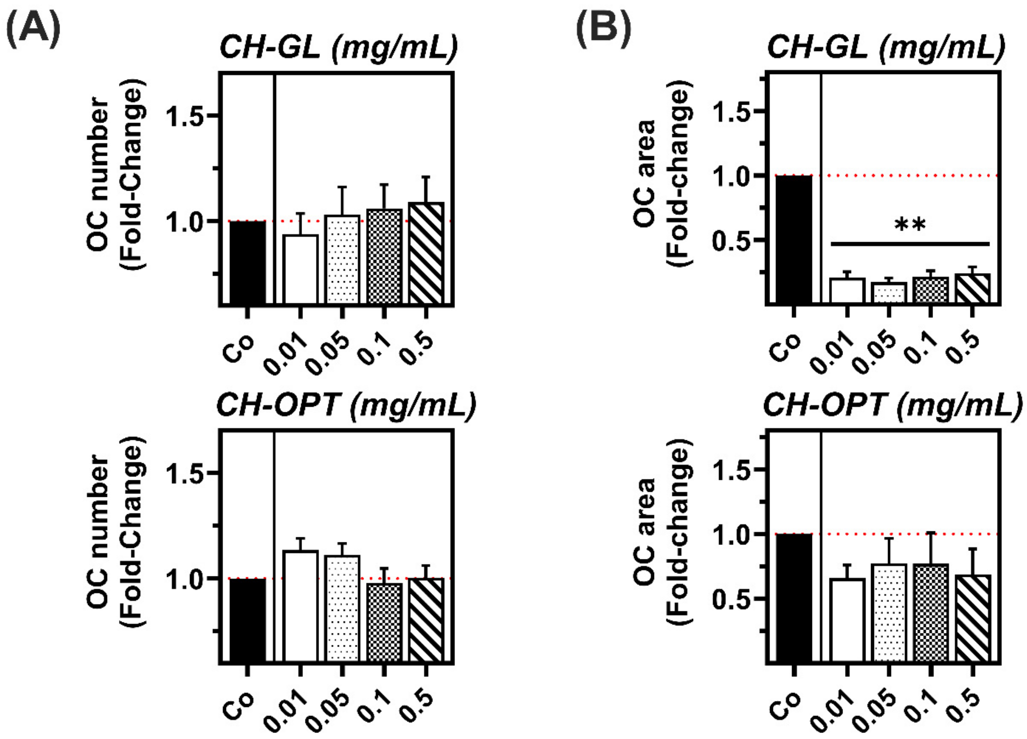

Figure S2. Exploratory analysis of the effects of CH digesta treatment on OC number and area under enhanced osteoclastogenic stimulation (RANKL, 100 ng/mL). Bone marrow-derived monocytes were differentiated into mature TRAP-positive OCs under high RANKL conditions (M-CSF, 50 ng/mL; RANKL, 100 ng/mL), with or without CH digesta treatment. (A) Average number of differentiated OCs and (B) average area per mature OC (µm²) at different CH digesta concentrations, expressed as fold-change relative to the corresponding differentiation control (Co). Data are presented as mean ± standard error of the mean (SEM). Because OC number and area values were normalized to the corresponding differentiation control, which was set to 1.0, each treatment group was compared with the hypothetical value of 1.0 using a two-tailed one-sample ratio t-test or a Wilcoxon signed-rank test, as appropriate. Statistical annotations indicate treatment-versus-control comparisons. For OC number, $n = 8$ biological replicates evaluated using three technical replicates per donor; for OC area, $n = 5$ biological replicates representing independent animal donors; $^{**}p < 0.01$.

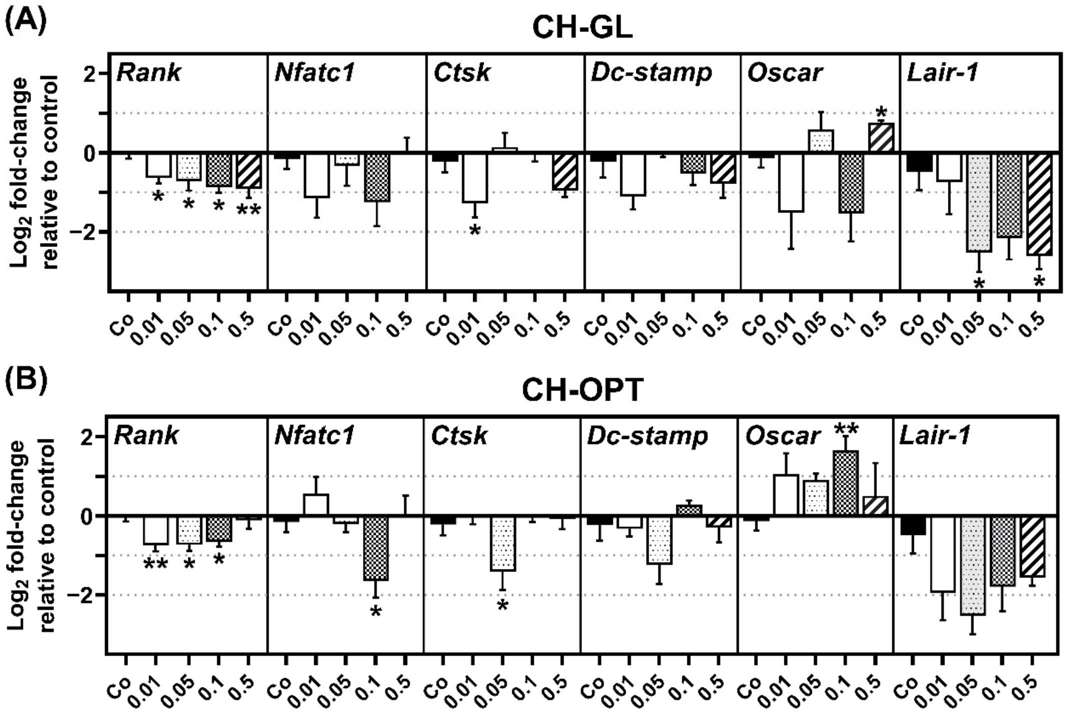

Figure S3. Exploratory analysis of CH digesta effects on osteoclastogenic gene expression under enhanced osteoclastogenic stimulation (RANKL, 100 ng/mL). Gene expression levels were assessed in OCs treated with (A) CH-GL or (B) CH-OPT (0.01, 0.05, 0.1, or 0.5 mg/mL) under high RANKL conditions. Data are presented as log_2-transformed fold-change relative to the corresponding differentiation control (Co) and reported as mean ± SEM. Statistical significance was determined using ordinary one-way ANOVA followed by Dunnett's multiple-comparisons test, Welch's ANOVA followed by Dunnett's T3 multiple-comparisons test, or the Kruskal–Wallis test followed by Dunn's multiple-comparisons test, as appropriate. Statistical annotations indicate treatment-versus-control comparisons. The analysis included *n* = 5–8 biological replicates per group, evaluated using two technical replicates per donor; *$p < 0.05$, **$p < 0.01$.
